# Bilateral Facial Diplegia: A Rare Presenting Symptom of Lyme

**DOI:** 10.1155/2017/4521526

**Published:** 2017-03-16

**Authors:** John Ashurst, Matthew Perry

**Affiliations:** Duke Lifepoint Conemaugh Memorial Medical Center, Department of Emergency Medicine, Johnstown, PA, USA

## Abstract

Lyme disease is a common disease that is faced by the physician but also acts a mimicker of many other disease processes. Facial palsies, especially bilateral, are a relatively rare presenting symptom of Lyme disease and may warrant further investigation. A thorough history and physical examination coupled with precision testing may aid the physician when faced with a patient with the diagnostic dilemma of facial diplegia.

## 1. Introduction

Lyme disease is the most common tick borne illness in the United States with an estimated 36,000 reported cases annually [1]. Although a large number of cases are reported to the Center for Disease Control, more than 300,000 cases remain unreported each year [1]. The most common sign of the disease is a rash known as erythema migrans, which may occur up to 1 to 2 weeks after infection but has been seen up to 4 weeks later [2]. Unlike the commonality of the “bulls eye rash” of Lyme disease, bilateral facial palsy is a relatively rare presenting symptom of the disease. The authors report a case of Lyme disease that presented with two-week history of facial swelling and facial diplegia.

## 2. Case

A 21-year-old male presented to the emergency department with a 2-week history of facial swelling and now decreased facial movement for the last several days. He was recently seen by his dentist and placed on clindamycin for a presumed dental infection several days prior to presentation but notes that the symptoms have worsened. Currently, he notes that he has a difficult time opening and closing his mouth but denies any difficulty with mastication. His past medical history was negative and his social history noted that he recently returned from a camping trip in western Pennsylvania.

**Figure 1:**
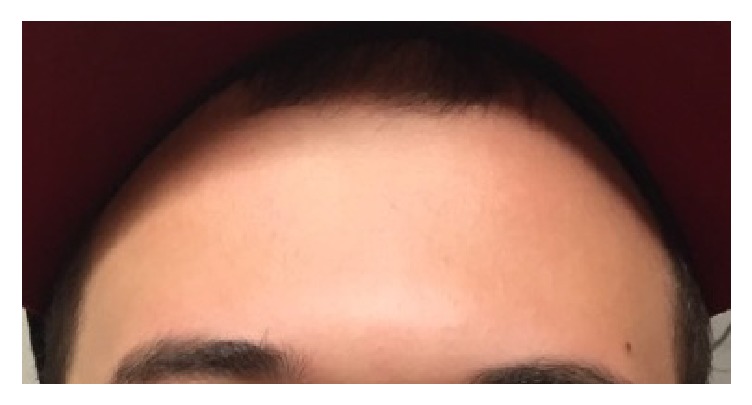
Bilateral loss of forehead creases while attempting to elevate the eyebrows.

**Figure 2:**
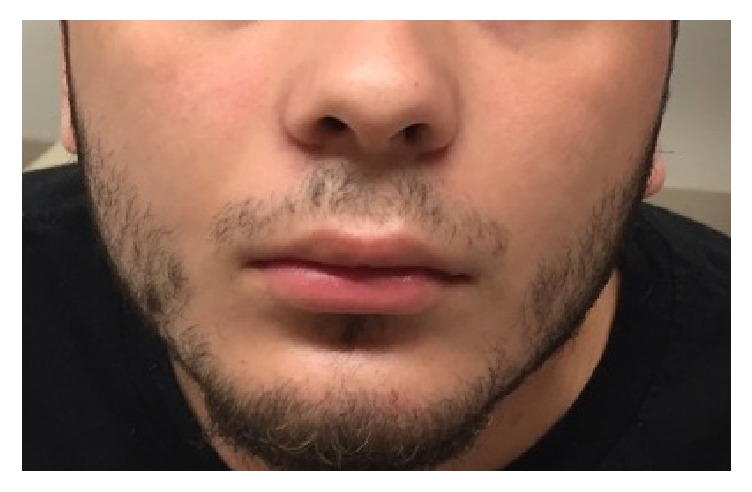
Patient attempting to smile with loss of cranial nerve VII bilaterally.

On physical exam, vital signs were within normal limits. He was able to open and close his mouth but noted that he could not open to the full extent. He had no drooling or changes in phonation. Upon examination of his teeth, he had dental fillings but no evidence of dental abscess, dental discomfort, or gingival abscess. A neurological exam revealed a normal gait and strength. He stated that he had a difficult time raising his eyebrows (Figure 1) and was only able to make a very small smile (Figure 2). He also had subjective sensory loss to light touch over the cheeks bilaterally but this could not be reproduced on examination. Besides the bilateral peripheral nerve palsy, the remainder of his neurological examination was with in normal limits.

Labs revealed a normal complete blood count, basic metabolic panel, and sed rate. Rapid Lyme titers were sent and revealed a positive Lyme IgM and IGG titer (Vidas Lyme IGG II and IgM II, Biomerieux). The patient was started on doxycycline for 21 days and a confirmatory western blot was sent out to a reference lab. The western blot confirmed that the patient was Lyme IgM positive, which was consistent with early infection of* Borrelia burgdorferi*.

## 3. Discussion

Bilateral facial paralysis (diplegia) is a relatively rare condition and comprises roughly 0.3% to 2% of all facial palsies [3]. In order to be described as a simultaneous facial palsy, instead of a recurrent unilateral facial palsy, the patient must present with opposite sided facial symptoms within 30 days of the onset of the first side. The differential diagnosis of patients with facial diplegia is broad and should include infection, trauma, metabolic, neoplastic, autoimmune, neurological, and idiopathic causes [4]. However, several studies have noted that Lyme disease is one of the most common causes of facial diplegia [4, 5].

Lyme disease is caused by the spirochete* Borrelia burgdorferi* and is transmitted through the bite of infected* Ixodes ricinus* ticks. Neurological manifestations can occur in up to 15% of patients and the classic triad of lymphocytic meningitis, cranial neuritis, and radiculoneuritis is a hallmark of this level of infection [6]. Cranial neuritis most commonly involves the seventh cranial nerve but can also involve cranial nerves involved in extraocular eye movements, hearing, and sensation of the face [6]. Classically, when cranial nerve VII is involved the patient will present with acute onset of one sided facial weakness, but approximately 25% of cases are now being reported as having bilateral diplegia [6].

**Figure 3:**
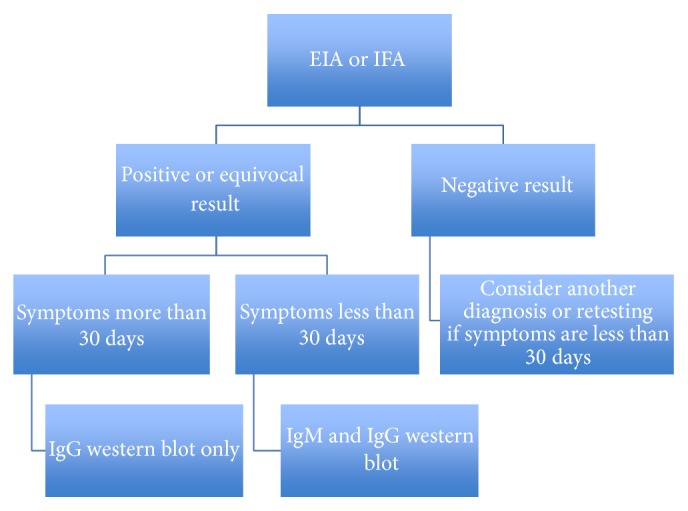
A step-wise approach for the diagnosis of* Borrelia* infection in a patient with clinically suspected Lyme disease.

The standard of care for diagnosing disseminated Lyme disease is a two-tiered serologic approach that garners approximately 70–100% sensitivity and a specificity of approximately 95% [7]. The first tier involves either enzyme immunoassay (EIA) or an immunofluorescence assay (IFA) measuring the overall antibody response to either IgM or IgG [7]. If either of these results is equivocal or positive, an immunoblot should be used to detect antibodies against* B. burgdorferi *(Figure 3) [7]. The immunoblot detects antibody reactivity to specific preselected* B. burgdorferi* antigens and is considered positive if bands are equal or greater than the intensity of the control [7].

Currently, the Infectious Disease Society of America has published guidelines on the treatment of Lyme disease and is based upon diagnosis. For those with cranial nerve palsies without clinical evidence of meningitis, doxycycline, amoxicillin, and cefuroxime are all considered appropriate treatment modalities [8]. Although doxycycline should be considered first-line therapy due to its penetrance into the central nervous system, the physician should tailor antibiotic choice to the patient's age and allergies [8].

## 4. Conclusion

In conclusion, those who present to the physician with facial diplegia represent a diagnostic dilemma due to both the relative scarcity of this presenting symptom and the morbidity of the diseases in the differential. However, those who live in endemic areas for Lyme disease should be aware of the correlation between facial diplegia and the disease. A thorough history and physical exam may aid the physician in diagnosis and allow for prompt treatment in order to prevent long term sequelae.
